# Impact of accurate initial discharge planning and inpatient transfers of care on discharge delays: a retrospective cohort study

**DOI:** 10.1136/bmjopen-2024-097563

**Published:** 2025-05-30

**Authors:** Dan Burns, Chris Duckworth, Carlos Lamas-Fernandez, Rachael Leyland, Mark Wright, Matt Stammers, Michael George, Michael Boniface

**Affiliations:** 1 School of Electronics and Computer Science, University of Southampton, Southampton, UK; 2 Southampton Business School, University of Southampton, Southampton, UK; 3 University Hospital Southampton Foundation Trust, Southampton, UK

**Keywords:** Hospitals, Decision Making, Organisation of health services, Risk management, Electronic Health Records

## Abstract

**Abstract:**

**Objective:**

To investigate the association between initial discharge planning and transfers of inpatient care with discharge delay. To identify operational changes which could expedite discharge within the Discharge to Assess (D2A) model.

**Design:**

Retrospective cohort study.

**Setting:**

University Hospital Southampton National Health Service Foundation Trust (UHS).

**Participants:**

All adults (≥18 years) who registered a hospital inpatient stay in UHS between 1 January 2021 and 31 December 2022 (n=258 051). After excluding inpatient stays without complete discharge planning data or key demographic/clinical information, 65 491 inpatient stays were included in the final analysis. Data included demographics, comorbidities, ward movements, care team handovers and discharge planning records.

**Primary and secondary outcome measures:**

The primary outcome was discharge delay, defined as the number of days between the final estimated discharge date and the actual discharge date. For the purposes of OR analysis, discharge delay was modelled as a binary outcome: any delay (>0 days) versus no delay. Logistic regression models were used to examine associations between initial discharge planning accuracy, the number of ward moves and the number of in-specialty handovers and the likelihood of discharge delay, adjusting for demographic and patient complexity factors.

**Results:**

Out of 65 491 inpatient stays, 10 619 had an initial planned discharge pathway that was different from the final discharge pathway. 7790 of these inpatient stays (75.1%) recorded a discharge delay. In contrast, among the 54 872 inpatient stays where the initial and final pathway matched, 10 216 (18.6%) recorded a delay. Using logistic regression modelling a binary outcome (any discharge delay vs no delay), an inaccurate initial pathway was associated with significantly increased odds of delay (adjusted OR (aOR) 2.72, 95% CI 2.55 to 2.91). Each additional ward move (aOR 1.25, 95% CI 1.23 to 1.28) and each in-specialty handover (aOR 1.17, 95% CI 1.14 to 1.20) were also associated with higher odds of discharge delay.

**Conclusions:**

This study finds a strong association between inaccurate initial discharge plans and inpatient transfers of care with discharge delay, after controlling for patient complexity and acuity. This highlights the need to consider how initial plans and inpatient transfers affect discharge planning. Given the lead times for organising onward care, operational inefficiencies are most impactful for patients eventually discharged on pathways with higher planning complexity.

**STRENGTHS AND LIMITATIONS OF THIS STUDY**

- Used a large, routinely collected dataset from a major National Health Service hospital, ensuring comprehensive capture of demographic, clinical and operational variables.
- Developed a novel binary metric for discharge planning accuracy based on initial and final pathway alignment within the Discharge to Assess policy framework.
- Excluded many short-stay inpatient stays due to missing discharge planning data, potentially limiting generalisability to non-complex cases.
- Study design was single-site and retrospective, which may affect the transferability of findings and introduce selection bias.

## Introduction

 The UK National Health Service (NHS) is under the most severe pressure in its 75-year history. In NHS England alone, 7.6 million patients are waiting for treatment, with up to 2.5 million patients waiting more than a year for elective care.^1^ Bed availability remains at critical levels, with occupancy consistently exceeding 93% through winter 2023/2024.^2^ Contributing to this pressure is the large number of patients remaining in hospital despite no longer meeting the criteria to reside. As of January 2024, 14 436 patients a day on average remained in hospital despite being ready to leave, with delays to discharge steadily rising over the last 3 years (30% higher relative to December 2021).^3^

Delays to discharge lead to worse outcomes for patients, including physical and cognitive deconditioning and increased risk of hospital-acquired infection.^4^,^8^ Delays also imperil other patients who either experience prolonged stays in the emergency department or are unable to be admitted for urgent elective care.^4 9^ Despite 85% of hospital inpatients being discharged without additional support, the most common reason for discharge delay is often due to limited availability of post-acute or community-based care services (over 65% of all inpatient stays),^7^ that is, support services provided outside of the hospital setting, such as home care, rehabilitation or placement in long-term care facilities. The transfer from hospital to post-acute or community-based care can be complex. It can be delayed due to capacity problems in the workforce and onward care services, lack of patient information to plan onward care and inefficiencies in communication or the discharge process. For patients in need of onward care, any delay in assessment, choice or access prevents discharge from hospital.^6^

Discharge planning, designed to mitigate delays to discharge, is the process of setting up an onward package of care for patients in the hospital. This requires the hospital care team to assess and predict the needs of the patient pretreatment, during treatment and post-treatment. With post-acute and community-based care capacity issues causing a significant lead time on care organisation, delays to discharge are possible if plans cannot be made in a timely manner. Despite this, patient complexity (eg, age, comorbidities, number of episodes and ward/specialty transfers) greatly impedes clinical ability to make informed and timely predictions of onward care, particularly when the patient’s condition or care needs change abruptly during a hospital inpatient stay. Furthermore, planned discharge pathway changes throughout the patient’s stay result in a need to restart the planning process, potentially leading to delays in discharge.

Despite the large-scale impact of discharge delay on the NHS,^10 11^ few studies have been conducted to understand its drivers in a hospital setting and are often targeted at specific patient subgroups such as vascular surgery,^12^ hip fractures,^13^ paediatric intensive care^14^ and trauma^15^ patients. One study has been carried out for admission from an emergency department finding that demographic and arrival mode are determinants of a delayed transfer of care.^16^ Meta-analyses on the impact of discharge planning on length of stay have been carried out and reveal small reductions in length of stay overall.^17 18^ Despite this, there is a notable lack of standardisation in the definition of discharge planning.^19^ A robust definition of planning quality is therefore missing.

In this study, we define a way to measure initial planning quality through the Discharge to Assess (D2A) model of care.^20 21^ Using this, we investigate how discharge delay varies by initial discharge planning, care organisation through the inpatient stay and inpatient complexity. We look to quantify how the accuracy of initial planning (ie, the ability to predict onward care needs) impacts the likelihood and magnitude of discharge delay. We also consider operational circumstances (eg, transfer of care between clinical teams or ward changes) and patient complexity to quantify factors which significantly reduce clinical ability to create accurate onward care plans and how these complex care needs may be harder to organise.

## Methods

### Data sources and study design

This cohort study is based on routinely collected data at University Hospitals Southampton NHS Foundation Trust (UHS). Information, including patient demographics and inpatient stays, was extracted directly from the UHS’s systems. Inpatient stay data detail the admission date, discharge date, consultant episodes within the inpatient stay, the consultant’s specialty and International Statistical Classification of Diseases and Related Health Problems, 10th Revision (ICD10) codes associated with the inpatient stay.^22^ Each inpatient stay is made up of one or more consultant episodes, which is defined as a continuous period where one or more consultants within a particular specialty have leadership over the care of the patient. Each episode contains information about the length of the episode, the associated care specialty and ICD10 codes recording the reason for the stay and any comorbidities or complications that occurred during the episode. Each inpatient stay has a dominant episode (and therefore dominant specialty of care), which is defined as the most resource-intensive episode by the NHS Healthcare Resource Grouper.^23^ The database also contains information about which wards the patient stayed in during their inpatient stay.

Discharge planning information is extracted from the UHS’s Application Express (APEX) system. APEX is an audit log of changes to each patient’s discharge plan with associated timestamps. It contains information about their discharge pathway as assessed by the discharge team, estimated discharge date (EDD) and identified onward care needs. The pathways are characterised by the corresponding numeric pathway in the UK’s D2A model of care.^20 21^ In this care model, organisation of onward care, ranging from short-term rehabilitation and reablement to more permanent long-term arrangements, is organised into four discharge pathways:

- *Pathway 0* reflects a simple discharge case or a restart of existing care packages.
- *Pathway 1* involves discharge to the usual place or residence with additional support, such as reablement, therapy or longer-term at-home packages of care.
- *Pathway 2 *involves temporary bed-based settings such as in-patient rehabilitation, mental health or hospice beds with short-term service or respite placements.
- *Pathway 3* indicates a permanent care home or long-term bed-based care setting, typically a new admission to a long-term care facility or home or a return to a pre-existing care home placement.

The pathway reflects a scale of planning complexity: from pathway 0 with minimal planning, to pathway 3 having the highest planning complexity for patients with multiple onward care needs. As the discharge planning process evolves, the pathway and EDD fields are updated and timestamped accordingly. Therefore, there can be multiple pathways and EDD reassessments in each inpatient stay record. The final recorded D2A pathway represents the actual route through which the patient was discharged, with the final EDD representing the date that a patient is due to be medically fit for discharge.

### Study population

We include all inpatient stays concerning adult patients (≥18 years old) between 1 January 2021 and 31 December 2022. The inclusion criteria were inpatient stays: (1) that had discharge planning information recorded, (2) when the patient is alive at the point of discharge and (3) that had complete demographics and ICD10 codes. We exclude inpatient stays which had a rare dominant specialty or admission type, that is, when there were fewer than 100 inpatient stays with that dominant specialty or admission type.

### Outcome measurement

The primary outcome of interest was whether a discharge delay occurred during an inpatient stay, defined as a binary variable: delayed (any delay >0 days) or not delayed (no delay). Discharge delay was determined by comparing the actual discharge date to the final EDD recorded by the care team. If the actual discharge date was later than the EDD, the inpatient stay was classified as delayed. For inpatient stays where the EDD was after the discharge date or no delay occurred, the outcome was classified as not delayed. For inpatient stays where the EDD was updated on the day of discharge, the second-to-last recorded EDD was used when available to avoid artificially recording no delay. If no earlier EDD was available, the inpatient stay was classified as not delayed.

### Covariates

To describe patient complexity, we extract the number of comorbidities based on those used in the Charlson Comorbidity Index from each inpatient stay. We also extract the specialty of the dominant episode (henceforth referred to as the dominant specialty), the number of unique specialities involved in an inpatient stay and whether the inpatient stay was elective. The age of the patient at hospital admission is also extracted. We also extract whether the inpatient stay involved a stay in an intensive care unit (ICU) from the ward stays data.

To describe operational and planning complexity, we extract the number of ward moves a patient undertakes during an inpatient stay and the number of times an inpatient stay contained an in-specialty handover between consultant teams. An in-speciality handover is defined as a transfer of care responsibility between consultants of the same specialty (eg, a consultant in general medicine transferring the patient’s care to another consultant in general medicine).

To describe initial planning, we extract the initial and final recorded D2A pathway and create a binary variable indicating whether these pathway records match. In cases where there was only one pathway entry for the patient, the initial pathway and final pathway match by default.

### Statistical analyses

We first calculated descriptive statistics for each covariate category within the final study cohort, stratified by whether a discharge delay occurred. To assess potential cohort selection effects, we also compared the length of stay distributions between included inpatient stays and those excluded due to missing discharge planning data.

To estimate the association between operational factors and discharge delay, we performed logistic regression analyses. Three models were specified for each operational variable of interest: whether the initial discharge pathway matched the final pathway, the number of ward moves and the number of in-specialty handovers. The first model was unadjusted, estimating the crude association with discharge delay. The second model adjusted for operational factors only, incorporating all three operational variables along with the final discharge pathway. The third and fully adjusted model included additional covariates reflecting patient complexity: age, number of comorbidities, dominant specialty, elective status, ICU stay and the number of specialties involved.

The primary aim of this modelling strategy was to minimise confounding bias when estimating the independent effects of planning accuracy and operational factors on discharge delay. Covariates for adjustment were selected a priori based on their established association with patient complexity and discharge outcomes in the prior literature.

Model performance was evaluated using the area under the receiver operating characteristic curve (AUROC) with 95% CIs estimated by bootstrapping to evaluate explanatory power, but model selection was not driven by maximising predictive performance. The AUROC was used to evaluate whether the model provided adequate explanatory power to distinguish between inpatient stays with and without discharge delay. If model discrimination had been found to be poor (AUROC <0.7), we would have either reviewed covariate selection, explored whether additional relevant confounders or interaction terms should be included to improve model fit or not published the analysis.

To account for potential temporal variation due to changes in hospital operations during the COVID-19 pandemic, we stratified the analyses by year of admission (2021 and 2022). Analyses were performed using Python V.3.9.5.

### Subgroup and sensitivity analyses

To explore potential effect modification, we extended the fully adjusted model to include interaction terms between each operational variable and patient complexity covariates. Where interactions were statistically significant, we performed stratified analyses by relevant subgroups, including age, sex, number of comorbidities and final pathway. Interaction terms and stratified ORs are reported where applicable.

## Results

### Descriptive statistics

**Figure 1:**
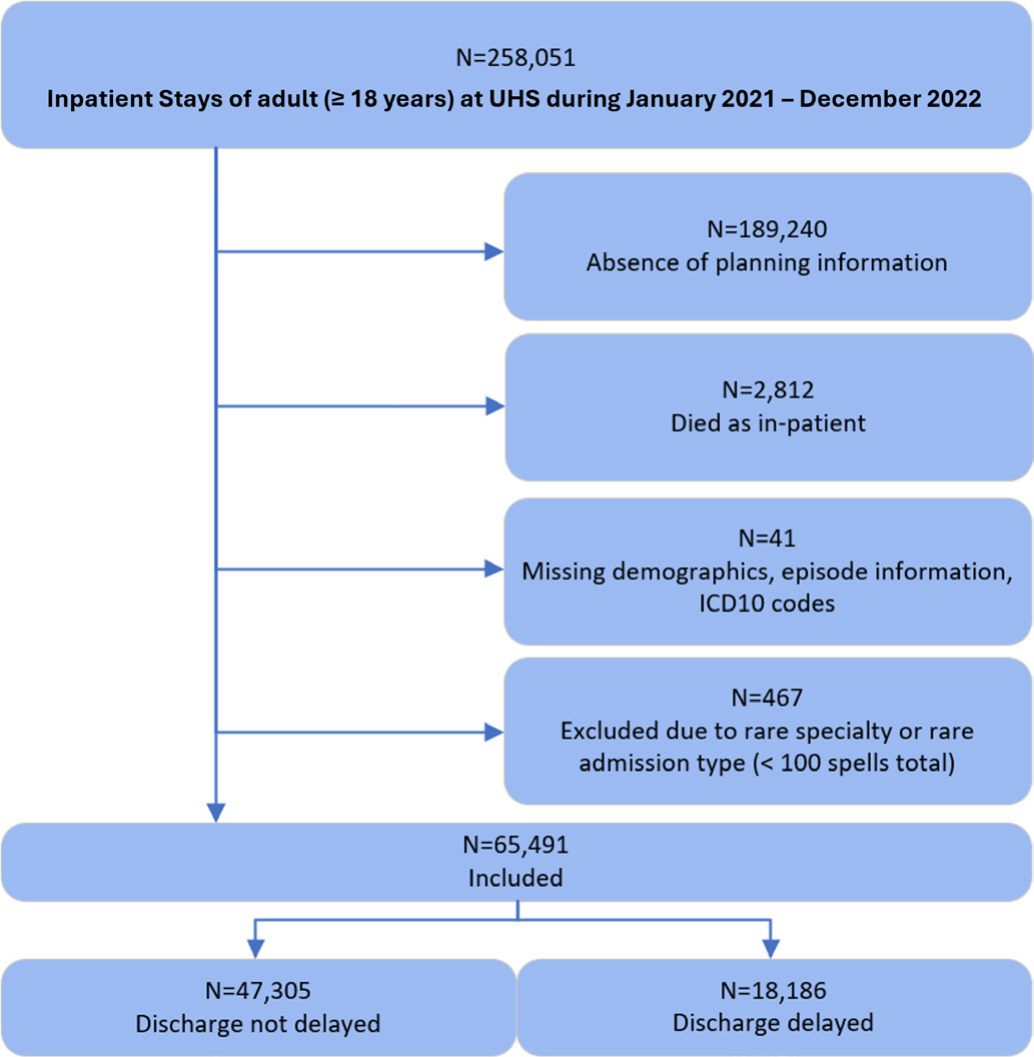
Cohort selection diagram during study period. ICD10, International Statistical Classification of Diseases and Related Health Problems, 10th Revision UHS, University Hospital Southampton National Health Service Foundation Trust.

From an initial sample of 258 051 inpatient stays, 65 491 met the inclusion criteria for analysis (figure 1). Most excluded inpatients had short stays and lacked discharge planning data. Among excluded inpatient stays, 84% had a length of stay less than 1 day, which, by definition, would not have a delay. A further 8% stayed between 1 day and 2 days, suggesting they were predominantly short, low-complexity admissions. The included cohort had substantially longer stays overall.

Table 1 provides descriptive statistics of the study cohort, stratified by the presence or absence of discharge delay. Inpatient stays with discharge delay were more common among older patients, non-elective admissions, those with multiple comorbidities and patients requiring complex discharge pathways or experiencing multiple ward moves and in-specialty handovers.

**Table 1:** Descriptive statistics of the cohort inpatient stays, stratified by whether a discharge delay occurred or not

Variable	Discharge delayed	
	No (n=47 305)	Yes (n=18 186)
Age at admission in years		
18–44	9389 (19.8)	1263 (6.9)
45–64	13 200 (27.9)	2913 (16.0)
65–84	19 547 (41.3)	8638 (47.5)
85+	5169 (10.9)	5372 (29.5)
Dominant specialty		
Accident and emergency	934 (2.0)	625 (3.4)
Adult cystic fibrosis	160 (0.3)	8 (0.0)
Cardiac surgery	1688 (3.6)	417 (2.3)
Cardiology	2736 (5.8)	602 (3.3)
Clinical haematology	1213 (2.6)	207 (1.1)
Clinical oncology	994 (2.1)	284 (1.6)
Colorectal surgery	758 (1.6)	131 (0.7)
Ear, Nose and throat	957 (2.0)	95 (0.5)
Gastroenterology	624 (1.3)	149 (0.8)
General medicine	12 367 (26.1)	3924 (21.6)
General surgery	3607 (7.6)	543 (3.0)
Geriatric medicine	4512 (9.5)	4544 (25.0)
Hepatobiliary and pancreatic surgery	626 (1.3)	96 (0.5)
Hepatology	166 (0.4)	52 (0.3)
Interventional radiology	1699 (3.6)	546 (3.0)
Medical oncology	1476 (3.1)	297 (1.6)
Nephrology	81 (0.2)	26 (0.1)
Neurology	1077 (2.3)	578 (3.2)
Neurosurgery	1724 (3.6)	457 (2.5)
Oral surgery	576 (1.2)	61 (0.3)
Respiratory medicine	557 (1.2)	256 (1.4)
Spinal surgery service	1142 (2.4)	341 (1.9)
Thoracic surgery	1678 (3.5)	280 (1.5)
Trauma and orthopaedic	3150 (6.7)	2780 (15.3)
Upper gastrointestinal surgery	269 (0.6)	42 (0.2)
Urology	1592 (3.4)	334 (1.8)
Vascular surgery	942 (2.0)	511 (2.8)
Final pathway		
0 Simple discharge/existing care package restart	41 124 (86.9)	6563 (36.1)
1 Discharge home with additional support	3724 (7.9)	4316 (23.7)
2 Temporary bed-based care (rehabilitation/respite)	1352 (2.9)	4450 (24.5)
3 Permanent care home or long-term placement	1105 (2.3)	2857 (15.7)
Initial pathway		
Correct	44 656 (94.4)	10 216 (56.2)
Incorrect	2649 (5.6)	7970 (43.8)
Non-elective inpatient stay		
No	10 302 (21.8)	1915 (10.5)
Yes	37 003 (78.2)	16 271 (89.5)
Number of CCI comorbidities		
0	24 958 (52.8)	6444 (35.4)
1	10 289 (21.8)	4370 (24.0)
2	6159 (13.0)	3314 (18.2)
3+	5899 (12.5)	4058 (22.3)
Number of in-specialty handovers		
0	43 963 (92.9)	15 362 (84.5)
1	2408 (5.1)	1889 (10.4)
2	626 (1.3)	596 (3.3)
3+	308 (0.7)	339 (1.9)
Number of specialties involved		
1	45 521 (96.2)	17 053 (93.8)
2	1574 (3.3)	937 (5.2)
3+	210 (0.4)	196 (1.1)
Number of ward moves		
0	29 352 (62.0)	6914 (38.0)
1	10 176 (21.5)	5697 (31.3)
2	4423 (9.3)	2945 (16.2)
3+	3354 (7.1)	2630 (14.5)
Sex		
Male	24 687 (52.2)	8540 (47.0)
Female	22 618 (47.8)	9646 (53.0)
ICU stay		
No	44 195 (93.4)	16 980 (93.4)
Yes	3110 (6.6)	1206 (6.6)

Values are inpatient stay count (percentage).

CCI, Charlson Comorbidity Index; ICU, intensive care unit.

**Figure 2:**
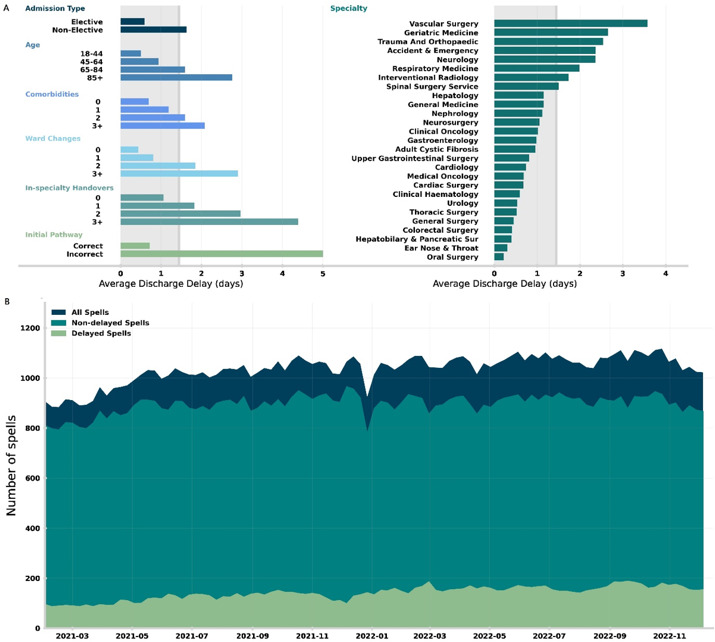
(**A**) Average discharge delay for inpatient stays between 01 January 2021 and 31 December 2022. The grey shading in the background gives the average discharge delay across all inpatient stays. Average discharge delay is then computed for inpatient stays grouped in different ways: (left, top to bottom) admission type, age at admission, number of registered comorbidities, number of ward changes during the inpatient stay, number of in-speciality handovers (ie, consultant handovers) and whether the initial pathway estimate was correct. (Right) Average discharge delay by dominant specialty, only including specialities with at least 100 registered inpatient stays. (**B**) Weekly average of the number of in-patients between 01 January 2021 and 31 December 2022, showing number of in-patients who are delayed with patients who are not delayed.

In the top panel (A) of figure 2, we compute the average discharge delay stratified by different covariate values. We find strong correlations (of increasing delay) with non-elective inpatient stays, age, number of comorbidities, ward changes, in-speciality handovers and incorrect initial pathways. Vascular surgery, geriatric medicine and trauma and orthopaedics have the highest average delays when stratifying by care specialty, notably all specialities with high likelihood of complex onward care. In the bottom panel (B) of figure 2, we show the weekly average number of in-patients (dark blue), further stratifying on whether the inpatient stay had a discharge delay (light green) or not (teal). We note a general trend of both increased capacity and delayed inpatient stays across the study period (01 January 2021–31 December 2022).

### Likelihood of discharge delay

In logistic regression analyses, inaccurate initial discharge planning and inpatient transfers of care were significantly associated with discharge delay (table 2). In fully adjusted models, an incorrect initial discharge pathway nearly tripled the odds of delay (aOR 2.72, 95% CI 2.55 to 2.91). Each additional ward move increased the odds by 25% (aOR 1.25, 95% CI 1.23 to 1.28), and each in-specialty handover increased the odds by 17% (aOR 1.17, 95% CI 1.14 to 1.20). These effects persisted after adjusting for patient complexity, including age, comorbidities, dominant specialty, elective status, ICU stay and number of specialties involved. Model discrimination was good, with an AUROC of 0.823 (95% CI 0.818 to 0.827). Results remained consistent when stratified by admission year. Please see online supplemental table S1 for fully adjusted model ORs for each variable, online supplemental table S2 for the fully adjusted model stratified by final D2A pathway and online supplemental table S3 for the fully adjusted model stratified by age group.

**Table 2:** ORs between initial pathway different to final, number of ward moves and number of in-specialty handovers, with discharge delay for the crude, operationally adjusted and fully adjusted models

Variable	Total inpatient stays	OR			P value
		Model 1: crude	Model 2: operationally adjusted model	Model 3: fully adjusted model	
Initial pathway correct (n=54 872)	65 491	Reference	Reference	Reference	
Initial pathway incorrect (n=10 619)		13.15 (12.53 to 13.80)	2.87 (2.68 to 3.06)	2.72 (2.55 to 2.91)	<0.001
Stratified by time					
2021	32 968	13.18 (12.34 to 14.07)	2.03 (1.83 to 2.26)	1.92 (1.73 to 2.14)	<0.001
2022	32 523	13.82 (12.82 to 14.88)	2.58 (2.34 to 2.85)	2.46 (2.23 to 2.72)	<0.001
Secondary measures					
Number of ward moves (per move)	65 491	1.37 (1.35 to 1.39)	1.26 (1.24 to 1.28)	1.25 (1.23 to 1.28)	<0.001
Number of in-specialty handovers (per handover)	65 491	1.53 (1.50 to 1.56)	1.12 (1.09 to 1.15)	1.17 (1.14 to 1.20)	<0.001

ORs are quoted as the value of the OR with the 95% CI in brackets. Model 1 (crude): no covariate adjustment. Model 2 (operationally adjusted): adjusted for all three operational variables (initial pathway accuracy, number of ward moves, number of in-specialty handovers) and final discharge pathway. Model 3 (fully adjusted): adjusted for Model 2 covariates plus age, number of comorbidities, dominant specialty, elective status, ICU stay and number of specialties involved. All listed variables were included in the models regardless of statistical significance to minimise confounding bias. Only primary predictors of interest are reported in this table.

### Subgroup and sensitivity analyses

Interaction testing identified significant effect modification by age, elective status, ICU stay, number of specialties involved and final discharge pathway (all p<0.001). Stratification by D2A pathway showed the strongest effects of inaccurate initial planning among patients discharged on pathway 3 (OR 4.45, 95% CI 3.77 to 5.25), whereas those on pathway 2 were less affected (OR 1.82, 95% CI 1.54 to 2.14), with pathway 0 (OR 2.72, 95% CI 2.33 to 3.17) and pathway 1 (OR 2.57, 95% CI 2.30 to 2.87) remaining similar to the overall OR.

For stratification by age, patients were split into four groups for stratification analysis: 18–44 years, 45–64 years, 65–84 years and 85+ years. Patients in the 85+ years age group had the highest impact from an inaccurate initial pathway (OR 3.23, 95% CI 2.88 to 3.63), with the 45–64 years age group having the lowest impact (OR 1.82, 95% CI 1.50 to 2.20). Stratifications by the number of comorbidities and sex were not carried out as the interaction terms were insignificant.

For inpatient transfers of care, patients discharged on pathway 3 are most likely to be affected by ward moves (OR 1.41, 95% CI 1.30 to 1.52) and in-specialty handovers (OR 1.37, 95% CI 1.23 to 1.52). Pathway 1 patients were similar for both ward moves (OR 1.39, 95% CI 1.32 to 1.45) and in-specialty handovers (OR 1.39, 95% CI 1.30 to 1.48).

## Discussion

In this study, we found a strong association between the accuracy of an initial pathway assessment and discharge delay, with the odds of discharge delay increasing almost three-fold if the initial discharge pathway assessment is incorrect. We find that patients ultimately discharged on pathway 3 are most affected by inaccurate initial planning, with the odds of discharge delay increasing almost five-fold if the initial pathway is incorrect. However, it is important to acknowledge that discharge planning occurs in real time under considerable uncertainty, with evolving clinical conditions, unforeseen complications and changes in social or family circumstances often making initial plans appear inaccurate in hindsight. We also find that operational processes such as ward changes and in-speciality handovers (ie, a transfer of care between consultants of the same specialty) have a substantial impact on the likelihood of delay, with three ward changes or four in-speciality transitions doubling the odds of delay. The effects from ward changes and in-speciality handovers remain after correcting for in-patient complexity (eg, comorbidities, number of care specialities involved in treatment) and are strong for the most acute onward care needs (ie, pathway 3). As a result, those with the potentially highest acuity onward care needs should be prioritised when considering operational improvements (eg, reducing transfer of care).

Since pathway 3 is a permanent care placement, both local authorities and integrated care boards must be confident and assured that all other pathway options have been exhausted and that the patient’s needs are clear (including a robust mental capacity assessment and wishes are captured). As a result, pathway 3 requires the most extended lead times to organise onward care. This has been identified in different patient subgroups including surgical patients^24^ and trauma patients.^15^ Early and accurate identification of potential discharge needs provides discharge teams additional time to organise care and avoid delay. Despite this, early identification of onward discharge needs is fundamentally hard and is exacerbated by extended periods of high bed occupancy.

A significant bottleneck on patient flow through the NHS is the capacity to discharge patients with new onward care needs as they become medically fit. Delays in discharge can derive from inefficiencies in in-hospital processes (eg, inaccurate prediction of onward care need resulting or delays awaiting test results); however, they are exacerbated by the ability of over-capacity onward care services to accept additional patients. Community services face consistent issues with both staffing and funding, causing significant lead times for hospital care teams to be able to organise care, especially for care home placements.^10^ Despite this, internal processes within hospitals contribute to delay, which should be targeted to help improve patient flow. Efficient and accurate planning in the hospital enables organisation to start earlier and absorb the potential long lead times on care placement.

There are several possible mechanisms for why transfers of in-patient care contribute to discharge delay. When the transfer occurs, information surrounding the patient’s care must be transferred to the new ward or consultant from their previous care. Information transfer between care teams may often be imperfect, and lack of care continuity potentially contributes to certain information around discharge planning being lost. Specifically in the case of ward changes, system inertia may contribute where patients are transferred to other wards to free up space in acute wards for emergency department patients and, consequently, moved to a lower priority relative to other more unwell patients, leading to less focus on these patients around the initial expected discharge time. Furthermore, periods of increased pressure with high levels of bed occupancy reduce clinical time to accurately assess discharge requirements early and can cause displacement of patients under the same specialty across the hospital.^25^ Coupled with unexpected changes to patient conditions or complications from treatment, predicting complex care needs is intrinsically difficult. Clinical decision support tools (eg, machine learning algorithms) have the potential to help clinical teams better identify patients with complex onward care requirements early. In complementary work, we demonstrate the ability of an explainable machine learning model to help identify appropriate discharge pathways.^26^

### Strengths and limitations of this study

This study has several strengths. First, the data collection for discharge planning is large and robust due to its usage by complex discharge teams at UHS, which itself is the largest hospital (defined by number of beds) in NHS England. The data are digitised and used for national reporting, feeding directly into the statistical snapshot for acute discharge delays aggregated by NHS England. Consequently, the EDD and discharge pathway updates are accurately recorded. The inpatient stay data are also comprehensive as these data are routinely collected and used for national reporting. This study also benefits from direct collaboration between hospital care teams, discharge teams and researchers to provide different perspectives.

A limitation is that while our analyses included a large cohort, the exclusion of inpatient stays lacking discharge planning information may limit generalisability and introduce some selection bias. This primarily affected short-stay patients (length of stay <1 day in 84% of excluded cases), which by definition would not have a delay. While this suggests that many excluded patients had lower complexity and likely did not require discharge planning, the exclusion introduces some potential for selection bias. By focusing only on inpatient stays with recorded planning data, the study population over-represents patients with more complex care needs and longer hospital stays. As a result, the estimated associations between discharge planning accuracy, operational factors and discharge delay may be larger than would be observed in a general inpatient population. Moreover, missing data may occasionally reflect documentation inconsistencies or unrecorded planning in high-pressure settings, which could further bias effect estimates. Future work should consider strategies to handle missing discharge planning data, such as sensitivity analyses assuming different missing data mechanisms, or incorporating imputation methods where appropriate.

A further limitation is that while our models adjust for patient complexity and stratify by discharge pathway, we did not include length of stay directly as a covariate due to its overlap with the outcome of interest (ie, discharge delay). As a result, it is unclear whether handovers independently contribute to discharge delays or are simply more common in patients who stay longer due to clinical complexity. Further analysis is warranted to disentangle whether handovers exert an independent effect on discharge delays or primarily reflect the extended stays required for patients with complex needs.

As with any observational study, there is a risk of residual confounding despite adjustment for major patient complexity and operational factors. While models included age, comorbidities, specialty, elective status, ICU stay and number of specialties, unmeasured factors such as staffing levels, weekend admissions or care team workload may influence both discharge planning accuracy and delays. However, given the comprehensive covariate adjustment and consistent findings across subgroups, the risk of severe confounding bias is judged to be moderate.

Other limitations include that this study is single site (ie, has no other site validating results) and discharge planning in other sites may differ.

## Conclusions

We find that inaccurate initial discharge plans are a significant factor in increasing the likelihood of discharge delay. However, initial planning must be understood within the context of real-time clinical care, where uncertainty is common and evolving patient needs, complications or social factors can lead to necessary changes in the discharge pathway. Clinical decision support tools to correctly assign patients to a discharge pathway may help with accurate and timely initial assessment and may be more important than predicting length of stay. Patients at high risk of requiring complex onward care needs (ie, short-term and long-term intensive care) should be a primary target for improving patient flow due to in-hospital delays in assessment and arrangement of onward care. Patients with complex needs should, wherever possible, remain on a single ward under the same team since these significantly impact the OR of a delayed discharge.

## Supplementary material

10.1136/bmjopen-2024-097563 online supplemental file 1

## Data Availability

Data are available upon reasonable request.
