# Supplementary material for: Impact of accurate initial discharge planning and inpatient transfers of care on discharge delays: a retrospective cohort study
Source: BMJ Open. 2025 May 30;15(5):e097563. doi: 10.1136/bmjopen-2024-097563 (PMC12128396; doi:10.1136/bmjopen-2024-097563)
Supplement: Supplementary file 2 [file bmjopen-2024-097563.reviewer_comments.pdf]

PEER REVIEW HISTORY

BMJ Open publishes all reviews undertaken for accepted manuscripts. Reviewers are asked to complete a checklist review form and are provided with free text boxes to elaborate on their assessment. These free text comments are reproduced below.

ARTICLE DETAILS

Title (Provisional)

Impact of accurate initial discharge planning and in-patient transfers of care on discharge delays: a retrospective cohort study

Authors

Burns, Daniel Kevin; Duckworth, Chris; Leyland, Rachael; Wright, Mark; George, Michael; Boniface, Michael; Lamas-Fernandez, Carlos; Stammers, Matt

---

VERSION 1 - REVIEW

**Reviewer**	**1**
**Name**	**Newnham, Evan**
**Affiliation**	**Eastern Health**
**Date**	**24-Dec-2024**
**COI**	**None**

The introduction is well written - there are a few colloquial phrases that could do with being more generalised to an international audience (eg: 'trolley waits', 'spells'). Additionally, the phrase 'social care' is used - is this about community support or about bed-based services such as rehab or specialised geriatric management (I am sure this term is well understood by a UK audience but the wording could be broadened to appeal to a broader international reader)?

The methods section could be significantly reduced in word count if required. The pathways, for example, are well described in the relevant reference.

In the results, are the authors able to comment whether the number of in-specialty handovers was related to length of stay (one would expect LOS to be longer for those on pathway 3 and for the number of handovers to be related to this)?

In the discussion, it is stated that Pathway 3 is affected by inaccurate initial planning - whilst this might be true in hindsight, the planning may have been entirely accurate at the time and unpredictable factors (such as complications, change in social circumstances or family opinion) might be at play. This could be reworded in both the discussion and the conclusion to acknowledge the realtime challenges treating teams face.

**Reviewer**	**2**
**Name**	**Nazar, Hamde**
**Affiliation**	**Newcastle University**
**Date**	**20-Feb-2025**
**COI**	**None**

Many thanks for the opportunity to review this interesting study.

The introduction is clear and thorough and appropriately justifies the need for the study.

The methods were similarly clear and well articulated. Results were comprehensive. The discussion was concise and highlighted the key findings and their implications for practice.

I very much enjoyed reading this and gaining insight into how hospital data has been used to explore delays to discharge.

I have no concerns to raise about rigour or presentation. The authors are to be commended and I have no reservations to recommend for publication with no revisions.

**Reviewer**	**3**
**Name**	**Dack, Kyle**
**Affiliation**	**University of Bristol, MRC Integrative Epidemiology Unit**
**Date**	**10-Apr-2025**
**COI**	**None**

Statistical summary:

I have two relatively minor concerns. First, the modelling strategy is a little unclear – its not directly explained why covariates were adjusted for – and the reporting of the model results could also be clarified. Second, there are a lot of patients excluded due to missing data, and this could introduce selection bias. I think the authors could discuss this more in the limitations – it is reported as a limitation but the consequences on the study results are not really explored.

Abstract

1. Line 29. An odds ratio analysis is described – but what is the outcome for which odds were modelled? The primary outcome on line 28 is a continuous measure – the number of days discharge was delayed – so it is not clear what outcome was used for the odds model.

2. Line 35. "Out of 65,491 spells" from only reading the abstract this does not seem consistent with the participants subheading which describes including 258,051 participants

who had a hospital spell. The additional filtering/exclusions could be briefly described to explain how 65,000 was arrived at.

Introduction

3. No suggestions.

Methods

4. The primary outcome differs from that stated in the abstract. In the abstract, it is defined as a continuous measure of "number of days between the final estimated discharge date and the actual discharge date". Whereas in the outcome measurement section it is defined as a binary outcome of delayed or not delayed. The latter seems more accurate. While it is true that the binary outcome is derived from a continuous measure of days, I think it makes more sense to describe the variable as it was used in the analysis itself – which is binary.

5. Outcome measurements: related to the above, these two sentences seem to be somewhat contradictory "The primary outcome of interest is whether a discharge delay occurred within a given hospital spell", "we define discharge delay as the difference between the actual discharge date and the last reported EDD". I think the second sentence is not accurate – as stated at the end of the paragraph, discharge delay was defined as either true or false (binary).

6. The statistical analysis describes 3 models with different covariates but the rationale for the covariates isn't fully clear to me. Is the aim to maximise predictive power, or to have a set of covariates which minimize the risk of confounding bias in the estimates? I think this should be clarified because it is often a criteria on literature quality assessment tools.

7. "We check the model quality by assessing the Area Under the Receiver Operating

Characteristic curve (AUROC) to ensure that the model has adequate explanatory power with

confidence intervals calculated using bootstrapping" What did the authors do (or plan to do) if the power was not adequate?

Results

8. "Comparing to the chosen cohort, the 84th percentile of length of stay was 17 days, with the 92nd percentile" I could not follow the rationale for these percentiles. I understand it relates to the percentage of excluded spells, but its not clear what I should take from this or how they are comparable.

9. Table 1. If there is space, it would be really helpful for the pathways to have some kind of summary description so readers don't need to refer back to the methods to check how each is defined.

10. Table 2. Should the "total spells" for initial pathway correct/incorrect be divided to show the n for each group? This would be consistent with how time is presented.

11. Table 2. The covariate list for models 2 and 3 should be reported as footnotes. From this table, both model 2 and 3 seem to have identical covariates so its not clear where the difference is coming from. I am unsure if only significant results are being reported – but if so, this would not be transparent, and all predictors of interest should be reported However, this is not needed if covariates are only used to minimize confounding bias.

Discussion

12. Results are interpreted accurately and reasonably. I think that confounding should be discussed as a possible limitation – are there any factors which may influence both the predictors and odds of delay. The authors mention the high number of exclusions due to lack of planning information, and perhaps this could be one such factor. But I think it should be more directly discussed because it is a risk in any observational research so it would be helpful to read the authors judgement of how severe the risk of confounding bias is.

VERSION 1 - AUTHOR RESPONSE

Reviewer: 1

Dr. Evan Newnham, Eastern Health, Monash University

Comments to the Author:

The introduction is well written - there are a few colloquial phrases that could do with being more generalised to an international audience (eg: 'trolley waits', 'spells'). Additionally, the phrase 'social care' is used - is this about community support or about bed-based services such as rehab or specialised geriatric management (I am sure this term is well understood by a UK audience but the wording could be broadened to appeal to a broader international reader)?

Response: terms replaced by replaced by international known equivalents. "trolley waits" has replaced "prolonged stays in the emergency department". "Spells replaced by "inpatient stay". "Social care" replaced by "post-acute or community-based care services"

The methods section could be significantly reduced in word count if required. The pathways, for example, are well described in the relevant reference.

Response: The entire section has been refined to address reviewer comments.

In the results, are the authors able to comment whether the number of in-specialty handovers was related to length of stay (one would expect LOS to be longer for those on pathway 3 and for the number of handovers to be related to this)?

Response: Added paragraph to the limitation "A further limitation is that while our models adjust for patient complexity and stratify by discharge pathway, we did not include length of stay directly as a covariate due to its overlap with the outcome of interest (i.e., discharge delay). As a result, it is unclear whether handovers independently contribute to discharge delays or are simply more common in patients who stay longer due to clinical complexity. Further analysis is warranted to disentangle whether handovers exert an independent

effect on discharge delays or primarily reflect the extended stays required for patients with complex needs."

In the discussion, it is stated that Pathway 3 is affected by inaccurate initial planning - whilst this might be true in hindsight, the planning may have been entirely accurate at the time and unpredictable factors (such as complications, change in social circumstances or family opinion) might be at play. This could be reworded in both the discussion and the conclusion to acknowledge the realtime challenges treating teams face.

Response: added sentence to discussion "However, it is important to acknowledge that discharge planning occurs in real time under considerable uncertainty, with evolving clinical conditions, unforeseen complications, and changes in social or family circumstances often making initial plans appear inaccurate in hindsight."

Response: added sentence to conclusion "However, initial planning must be understood within the context of real-time clinical care, where uncertainty is common and evolving patient needs, complications, or social factors can lead to necessary changes in discharge pathway"

Reviewer: 2

Dr. Hamde Nazar, Newcastle University

Comments to the Author:

Many thanks for the opportunity to review this interesting study.

The introduction is clear and thorough and appropriately justifies the need for the study.

The methods were similarly clear and well articulated. Results were comprehensive. The discussion was concise and highlighted the key findings and their implications for practice.

I very much enjoyed reading this and gaining insight into how hospital data has been used to explore delays to discharge.

I have no concerns to raise about rigour or presentation. The authors are to be commended and I have no reservations to recommend for publication with no revisions.

Response: Thank you for comments.

Reviewer: 3

Dr. Kyle Dack, University of Bristol

Comments to the Author:

Statistical summary:

I have two relatively minor concerns. First, the modelling strategy is a little unclear – its not directly explained why covariates were adjusted for

Response: Added sentence to statistical analysis "Covariates were adjusted for to account for confounding factors such as age, comorbidities, and patient complexity, ensuring that

the associations between operational variables and discharge delay were not driven by underlying patient characteristics."

and the reporting of the model results could also be clarified.

Response: Reformatted the description of the models "To estimate the association between operational factors and discharge delay, we performed logistic regression analyses. Three models were specified for each operational variable of interest, whether the initial discharge pathway matched the final, the number of ward moves, and the number of in-specialty handovers. The first model was unadjusted, estimating the crude association with discharge delay. The second model adjusted for operational factors only, incorporating all three operational variables along with the final discharge pathway. The third and fully adjusted model included additional covariates reflecting patient complexity: age, number of comorbidities, dominant specialty, elective status, ICU stay, and the number of specialties involved."

Second, there are a lot of patients excluded due to missing data, and this could introduce selection bias. I think the authors could discuss this more in the limitations – it is reported as a limitation but the consequences on the study results are not really explored.

Response: Updated limitation section "A limitation is that while our analyses included a large cohort, the exclusion of inpatient stays lacking discharge planning information may limit generalisability and introduce some selection bias. This primarily affected short-stay patients (length of stay <1 day in 84% of excluded cases) which by-definition would not have a delay. While this suggests that many excluded patients had lower complexity and likely did not require discharge planning, the exclusion introduces some potential for selection bias. By focusing only on inpatient stays with recorded planning data, the study population over-represents patients with more complex care needs and longer hospital stays. As a result, the estimated associations between discharge planning accuracy, operational factors, and discharge delay may be larger than would be observed in a general inpatient population. Moreover, missing data may occasionally reflect documentation inconsistencies or unrecorded planning in high-pressure settings, which could further bias effect estimates. Future work should consider strategies to handle missing discharge planning data, such as sensitivity analyses assuming different missing data mechanisms, or incorporating imputation methods where appropriate."

Abstract

1. Line 29. An odds ratio analysis is described – but what is the outcome for which odds were modelled? The primary outcome on line 28 is a continuous measure – the number of days discharge was delayed – so it is not clear what outcome was used for the odds model.

Response: Updated results part of abstract "Out of 65,491 inpatient stays, 10,619 had an initial planned discharge pathway that different from the final discharge pathway. 7,790 of these inpatient stays (75.1%) recording a discharge delay. In contrast, among the 54,872 inpatient stays where the initial and final pathway matched, 10,216 (18.6%) recorded a delay. Using logistic regression modelling a binary outcome (any discharge delay vs no delay), an inaccurate initial pathway was associated with significantly increased odds of delay (adjusted odds ratio [aOR] 2.72, 95% CI 2.55–2.91). Each additional ward move (aOR

1.25, 95% CI 1.23–1.28) and each in-specialty handover (aOR 1.17, 95% CI 1.14–1.20) were also associated with higher odds of discharge delay."

Response: Clarified this in the primary and secondary outcome measures: "For the purposes of odds ratio analysis, discharge delay was modelled as a binary outcome: any delay (>0 days) versus no delay. Logistic regression models were used to examine associations between initial discharge planning accuracy, the number of ward moves, and the number of in-specialty handovers and the likelihood of discharge delay, adjusting for demographic and patient complexity factors."

2. Line 35. "Out of 65,491 spells" from only reading the abstract this does not seem consistent with the participants subheading which describes including 258,051 participants who had a hospital spell. The additional filtering/exclusions could be briefly described to explain how 65,000 was arrived at.

Response: Added exclusion to the participants section "After excluding spells without complete discharge planning data or key demographic/clinical information, 65,491 spells were included in the final analysis."

Introduction

3. No suggestions.

Methods

4. The primary outcome differs from that stated in the abstract. In the abstract, it is defined as a continuous measure of "number of days between the final estimated discharge date and the actual discharge date". Whereas in the outcome measurement section it is defined as a binary outcome of delayed or not delayed. The latter seems more accurate. While it is true that the binary outcome is derived from a continuous measure of days, I think it makes more sense to describe the variable as it was used in the analysis itself – which is binary.

Response: Updated outcome measurements section "The primary outcome of interest was whether a discharge delay occurred during a hospital spell, defined as a binary variable: delayed (any delay >0 days) or not delayed (no delay). Discharge delay status was determined by comparing the actual discharge date to the final estimated discharge date (EDD) recorded by the care team. If the actual discharge date was later than the EDD, the spell was classified as delayed. For spells where the EDD was after the discharge date, or no delay occurred, the outcome was classified as not delayed. For spells where the EDD was updated on the day of discharge, the second-to-last recorded EDD was used when available to avoid artificially recording no delay. If no earlier EDD was available, the spell was classified as not delayed."

5. Outcome measurements: related to the above, these two sentences seem to be somewhat contradictory "The primary outcome of interest is whether a discharge delay occurred within a given hospital spell", "we define discharge delay as the difference between the actual discharge date and the last reported EDD". I think the second sentence is not accurate – as stated at the end of the paragraph, discharge delay was defined as either true or false (binary).

Response: See above

6. The statistical analysis describes 3 models with different covariates but the rationale for the covariates isn't fully clear to me. Is the aim to maximise predictive power, or to have a set of covariates which minimize the risk of confounding bias in the estimates? I think this should be clarified because it is often a criteria on literature quality assessment tools.

Response: added modelling strategy to statistical analysis "The primary aim of this modelling strategy was to minimise confounding bias when estimating the independent effects of planning accuracy and operational factors on discharge delay. Covariates for adjustment were selected a priori based on their established association with patient complexity and discharge outcomes in prior literature. Model performance was evaluated using the AUROC (with 95% confidence intervals estimated by bootstrapping) to evaluate explanatory power, but model selection was not driven by maximising predictive performance."

7. "We check the model quality by assessing the Area Under the Receiver Operating

Characteristic curve (AUROC) to ensure that the model has adequate explanatory power with

confidence intervals calculated using bootstrapping" What did the authors do (or plan to do) if the power was not adequate?

Response: "The AUROC was used to evaluate whether the model provided adequate explanatory power to distinguish between inpatient stays with and without discharge delay. If model discrimination had been found to be poor (AUROC < 0.7), we would have either reviewed covariate selection, explore whether additional relevant confounders or interaction terms should be included to improve model fit or not publish the analysis"

Results

8. "Comparing to the chosen cohort, the 84th percentile of length of stay was 17 days, with the 92nd percentile" I could not follow the rationale for these percentiles. I understand it relates to the percentage of excluded spells, but its not clear what I should take from this or how they are comparable.

- Response: the percentile detail isn't critical, so we have streamlined the respond: "Among excluded inpatient stays, 84% had a length of stay less than one day, and a further 8% stayed between one and two days, suggesting they were predominantly short, low-complexity admissions. The included cohort had substantially longer stays overall."

9. Table 1. If there is space, it would be really helpful for the pathways to have some kind of summary description so readers don't need to refer back to the methods to check how each is defined.

Response: added pathway descriptions to Table 1

10. Table 2. Should the "total spells" for initial pathway correct/incorrect be divided to show the n for each group? This would be consistent with how time is presented.

Response: added sample sizes to Table 2

**Initial pathway correct (n = 54,872)**
**Initial pathway incorrect (n = 10,619)**

11. Table 2. The covariate list for models 2 and 3 should be reported as footnotes. From this table, both model 2 and 3 seem to have identical covariates so its not clear where the difference is coming from. I am unsure if only significant results are being reported – but if so, this would not be transparent, and all predictors of interest should be reported However, this is not needed if covariates are only used to minimize confounding bias.

Response: added explicit model 1,2, 3 with footnotes as follows "Model 1 (crude): no covariate adjustment. Model 2 (operationally adjusted): adjusted for all three operational variables (initial pathway accuracy, number of ward moves, number of in-specialty handovers) and final discharge pathway. Model 3 (fully adjusted): adjusted for Model 2 covariates plus age, number of comorbidities, dominant specialty, elective status, ICU stay, and number of specialties involved. All listed variables were included in the models regardless of statistical significance to minimise confounding bias. Only primary predictors of interest are reported in this table."

Discussion

12. Results are interpreted accurately and reasonably. I think that confounding should be discussed as a possible limitation – are there any factors which may influence both the predictors and odds of delay. The authors mention the high number of exclusions due to lack of planning information, and perhaps this could be one such factor. But I think it should be more directly discussed because it is a risk in any observational research so it would be helpful to read the authors judgement of how severe the risk of confounding bias is.

Response: Added general limitation with respect to this observational study "As with any observational study, there is a risk of residual confounding despite adjustment for major patient complexity and operational factors. While models included age, comorbidities, specialty, elective status, ICU stay, and number of specialties, unmeasured factors such as staffing levels, weekend admissions, or care team workload may influence both discharge planning accuracy and delays. However, given the comprehensive covariate adjustment and consistent findings across subgroups, the risk of severe confounding bias is judged to be moderate."

Reviewer: 1

Competing interests: Not applicable

Reviewer: 2

Competing interests: 'Not applicable'

Reviewer: 3

Competing interests: Not applicable
